# Comparison between an in‐house 1D profile correction method and a 2D correction provided in Varian's PDPC Package for improving the accuracy of portal dosimetry images

**DOI:** 10.1120/jacmp.v16i2.4973

**Published:** 2015-03-08

**Authors:** Maritza A. Hobson, Stephen D. Davis

**Affiliations:** ^1^ Department of Medical Physics McGill University Health Centre Montréal QC; ^2^ Medical Physics Unit, McGill University Montréal QC Canada

**Keywords:** EPID, portal dosimetry, calibration

## Abstract

While commissioning Varian's Portal Dose Image Prediction (PDIP) algorithm for portal dosimetry, an asymmetric radial response in the portal imager due to backscatter from the support arm was observed. This asymmetric response led to differences on the order of 2%–3% for simple square fields ($<20\times 20\;{\text{cm}}^{2}$) when comparing the measured to predicted portal fluences. A separate problem was that discrepancies of up to 10% were seen in measured to predicted portal fluences at increasing off‐axis distance (>10 cm). We have modified suggested methods from the literature to provide a 1D correction for the off‐axis response problem which adjusts the diagonal profile used in the portal imager calibration. This inherently cannot fix the 2D problem since the PDIP algorithm assumes a radially symmetric response and will lead to some uncertainty in portal dosimetry results. Varian has recently released generic “2D correction” files with their Portal Dosimetry Pre‐configuration (PDPC) package, but no independent testing has been published. We present the comparison between QA results using the Varian correction method to results using our 1D profile correction method using the gamma passing rates with a 3%, 3 mm criterion. The average, minimum, and maximum gamma pass rates for nine fixed‐field IMRT fields at gantry 0° using our profile correction method were 98.1%, 93.7%, and 99.8%, respectively, while the results using the PDPC correction method were 98.4%, 93.1%, and 99.8%. For four RapidArc fields, the average, minimum, and maximum gamma pass rates using our correction method were 99.6%, 99.4%, and 99.9%, respectively, while the results using the PDPC correction method were 99.8%, 99.5%, and 99.9%. The average gamma pass rates for both correction methods are quite similar, but both show improvement over the uncorrected results.

PACS numbers: 87.55.Qr, 87.55.N‐

## I. INTRODUCTION

In the past decade, several radiotherapy centers have started using portal imagers as a radiation dosimeter using different methods.^1^, ^2^, ^3^, ^4^, ^5^, ^6^, ^7^, ^8^, ^9^, ^10^, ^11^, ^12^, ^13^, ^14^ While commissioning the method available in our clinic (Varian's Portal Dose Image Prediction (PDIP) algorithm (Varian Medical Systems, Inc., Palo Alto, CA)), an asymmetric radial response in the portal imager due to backscatter from the imager arm was observed. It led to differences on the order of 2%–3% for simple square fields ($<20\times 20\;{\text{cm}}^{2}$) when comparing the measured to predicted portal fluences, which has also been noted by other authors.^15^, ^16^, ^17^, ^18^, ^19^, ^20^ However, the asymmetric response due to the backscatter from the arm is just one problem. The second problem, as discussed by Bailey et al.,^(21)^ is correcting for the off axis response of the detector (at distances>10 cm) so that the measured off axis response matches the predicted values provided by the PDIP.

The asymmetric and off axis problems are unacceptable for an accurate QA device, and one that will ultimately be used to collect portal dose images during treatment (transit or exit dosimetry images) and to compare these with predicted transit (exit) dosimetry images. Clinically significant differences between these two images could determine when replanning is necessary (i.e., if misalignments, patient motion, or weight changes make significant changes in the distribution). Having transit dosimetry images acquired during patient treatment would also increase the probability of detecting treatment errors, providing an additional patient safety measure. Mans et al.^(22)^ were able to use portal dosimetry to catch 17 serious treatment errors out of 4337 patients – nine of which would not have been identified using pretreatment testing methods. More recently Fuangrod et al.^(23)^ were able to use EPID imagers to catch real‐time errors in MUs, MLC leaf positions, and plan transfer.

Before accurate transit dosimetry images can be collected, both the asymmetric and off‐axis problems have to be corrected. Many authors have suggested methods to correct one or both of these problems by either changing the prediction model^16^, ^19^ or by modifying how the images are acquired.^15^, ^17^, ^18^, ^20^ Greer et al.^(16)^ provided a 2D image of the backscatter component of the Varian Exact arm that they determined from measurements with the EPID on and off the support arm. The simplest and most practical method within the clinical system to correct for the off‐axis response is the method provided by Bailey et al.,^(21)^ which is a 1D correction that adjusts the diagonal profile used in the detector calibration. This inherently cannot fix the 2D problem (i.e., the backscatter from the arm) since the PDIP algorithm assumes a radially symmetric response and leads to some uncertainty in portal dosimetry results. In the fall of 2012, Varian released generic “2D correction” files with their Portal Dosimetry Pre‐configuration (PDPC) package, which was recently described in detail by Van Esch et al.^(24)^ Unfortunately, the package is not available for all Varian linac models.^25^, ^26^

In this article, we present the results for different field types (simple square or rectangular fields, IMRT fields, and RapidArc fields) using a modified version of the method used in Bailey et al.^(21)^ and compare them to results collected using Varian's PDPC package in order to determine which method to use in our clinic.

## II. MATERIALS AND METHODS

### A. Equipment

All measurements were performed on the same day on a Varian linear accelerator with Millenium 120 MLCs and an amorphous silicon aS1000 EPID with a sensitive area of $40\times 30\;{\text{cm}}^{2}$ mounted on an ExactArm (Varian Medical Systems). All EPID images were acquired at the calibration location (isocenter) [(X,Y,Z)=(0,0,0)] with a matrix size of 512 pixels×384 pixels, giving a pixel pitch of 0.781 mm.

### B. Calibration and correction methods

Three different scenarios were used for calibrating the portal imaging system — one was using the standard method provided by Varian with no additional backscatter correction; the other used our modified 1D method for backscatter correction with two different food field sizes; the third used the Varian PDPC correction method. All calibrations were performed at isocenter (SID=100 cm).

Before any corrections for backscatter could be applied in the dose calibration step, dark field and food field calibrations needed to be performed. The dark field calibration provides a correction for background signal in the EPID by collecting image frames without any beam on, and the food field calibration uses an open beam to provide a sensitivity correction for the EPID. Greer^(27)^ provide a detailed explanation as to how the Varian software applies these two corrections to a raw image. The standard method provided by Varian used a food field size of $40\times 30\;{\text{cm}}^{2}$. Our modified 1D correction method also used a food field size of $40\times 30\;{\text{cm}}^{2}$, while the Varian PDPC correction method used a food field size of $40\times 32\;{\text{cm}}^{2}$.

Once the food field and dark field calibrations were complete, the dose calibration could be performed. During the dose calibration, the signal to the EPID for 100 MU from a $10\times 10\;{\text{cm}}^{2}$ field at the desired dose rate is collected and the image is renormalized such that one calibrated unit (CU) on a portal dosimetry image corresponds to the pixel value on the central axis after delivering 100 MU from a $10\times 10\;{\text{cm}}^{2}$ field. Since the food field divides out any nonuniformity in the photon field along with the variations in the detector sensitivity, a 1D profile correction is applied to the food field‐corrected and dark field‐corrected image to bring back the expected nonuniformity of the treatment beam (i.e., the “horns”). The standard Varian profile correction method uses a diagonal profile measurement in water from a $40\times 40\;{\text{cm}}^{2}$ field at $d_{\text{max}}$ in a 6 MV photon beam. Since our linac matches the Varian Golden Beam data within the measurement uncertainties, we used the diagonal profile provided with the Golden Beam data.

Bailey et al.^(21)^ modified the Varian standard method by applying a correction to the profile used during the dose calibration. This correction was determined by first performing the dose calibration using the Varian standard method, and then acquiring a portal dosimetry image of a $40\times 30\;{\text{cm}}^{2}$ field. Next, a diagonal profile from the center of the detector at a 45° angle was extracted for both the acquired and predicted portal dose images. The ratio of the profile from the measured portal dosimetry image to that of the predicted portal dosimetry image was used to create a correction to the values at the corresponding locations on the $40\times 40\;{\text{cm}}^{2}$ diagonal profile that was used in the dose calibration. This correction was determined from a first order (line) polynomial fit of the ratio versus diagonal distance from the origin from 10 to 25 cm.

**Figure 1:**
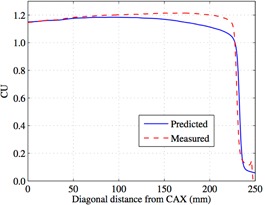
The diagonal profiles of the predicted and measured portal dosimetry images for a $38\times 28\;{\text{cm}}^{2}$ field without any correction.

Figure 1 shows the diagonal profiles of the measured and predicted portal dosimetry images for a $38\times 28\;{\text{cm}}^{2}$ field that we used to calculate the ratio for our correction. Bailey and colleagues^(21)^ stated that the ratio of the measured to predicted EPID signal below 10 cm was a nonlinear function of radial distance from central axis and needed a polynomial fit to achieve R^2^ above 0.99. Our method differs from that used in the Bailey study in that we use a fourth order polynomial and calculating this correction from central axis up to ~22.0 cm diagonally off axis, providing a correction factor ranging from −0.4% to 9.9% and an $R^{2}$ value of 0.999.

**Figure 2:**
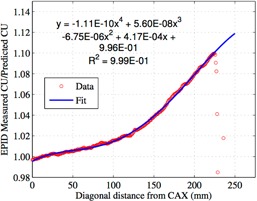
The ratio of the predicted to measured EPID signal and the polynomial fit used to generate the correction to the profile. The polynomial fit only uses data up to ~22 cm or else $R^{2}$ would not be 0.999.

**Figure 3:**
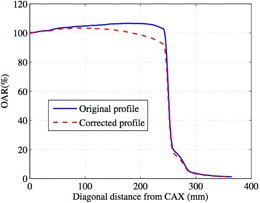
The corrected and uncorrected profiles used for dose calibration of the EPID for portal dosimetry.

Figure 2 shows the ratio of the measured‐to‐predicted EPID signal and the polynomial fit. We chose to use a $38\times 28\;{\text{cm}}^{2}$ field because this is approximately the maximum field size that we are interested in measuring. A second difference in our method from the Bailey method is that we also chose to fit only up to ~22 cm because past this is where the ratio of the measured to predicted EPID signal did not fit a fourth order polynomial, as can be observed in Fig. 2. This polynomial fit was used to correct the whole profile's data so that the whole imager could be used when collecting data. Figure 3 shows the corrected and uncorrected profiles that were used for our dose calibration.

Since a majority of our IMRT fields are smaller than $38\times 28\;{\text{cm}}^{2}$, we also investigated results from applying our profile correction method using a $20\times 20\;{\text{cm}}^{2}$ food field, which is a major difference from the Bailey et al.^(21)^ method, as well as the standard Varian method. The $20\times 20\;{\text{cm}}^{2}$ food field would include less backscatter contribution from the imager arm and, theoretically, should lead to reduced asymmetry in portal images for field sizes less than $20\times 20\;{\text{cm}}^{2}$. For this, we determined the correction for the profile by using the ratio of measured and predicted diagonal profiles from $20\times 20\;{\text{cm}}^{2}$ portal dose images. The polynomial fit used to determine the correction was fit up to ~11.0 cm. The correction was applied up to ~14.0 cm, which is the maximum diagonal for a $20\times 20\;{\text{cm}}^{2}$ field and, after this point, the correction was set to zero.

Another original part of this paper was to compare results using our correction method with results produced using the Varian PDPC method. Since this comparison has not been published before, we believe it will be useful to a typical clinical medical physicist when deciding whether or not to implement the Varian PDPC method or a simple 1D correction method. The Varian PDPC method provides one file to perform both its 2D correction for the backscatter from the imager arm and to bring back the beam profile in the portal dosimetry images that was divided out by the food field.^24^, ^25^, ^26^ Since our EPIDs are only licensed for half resolution, we had to use the file meant for the aS500 EPIDs (SID100_x06_40x32_aS500_PDPC1002.cdp).

### C. Plans tested

Once the imager was calibrated for a particular correction method, three different plan types were tested — square fields (from $5\times 5\;{\text{cm}}^{2}$ up to $28\times 28\;{\text{cm}}^{2}$), fixed‐field IMRT plans (23 fields at gantry 0° and at planned treatment angles), and two RapidArc plans. IMRT and RapidArc plans were tested since these are the type of plans that would typically need routine QA testing using the portal dosimetry method, whereas square fields would not. Plans were first calculated using Eclipse version 10.0 (Varian Medical Systems) with AAA version 10.0.28, and then predicted portal dose images were calculated using the Portal Dose Image Prediction (PDIP) version 10.0.28 for a delivery at isocenter (SID of 100 cm). All plans were set up using 6 MV photons.

## III. RESULTS & DISCUSSION

**Figure 4:**
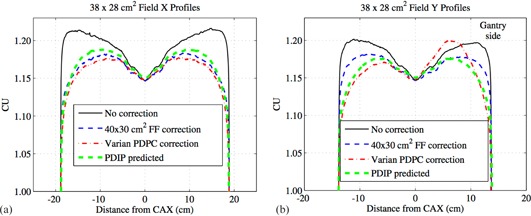
The X (transverse) profiles (a) from different correction methods for a $38\times 28\;{\text{cm}}^{2}$ field where the positive axis is towards the patient left side of the EPID. The Y (radial) profiles (b) from different correction methods for a $38\times 28\;{\text{cm}}^{2}$ field where the positive axis is towards the gantry side of the EPID.

**Figure 5:**
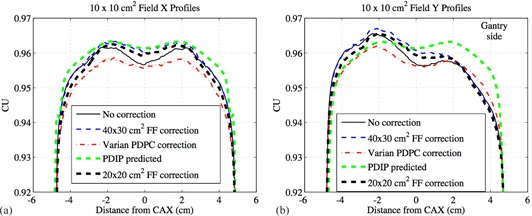
The X (transverse) profiles (a) from different correction methods for a $10\times 10\;{\text{cm}}^{2}$ field where the positive axis is towards the patient left side of the EPID. The Y (radial) profiles (b) from different correction methods for a $10\times 10\;{\text{cm}}^{2}$ field where the positive axis is towards the gantry side of the EPID.

Figures 4(a) and 4(b) present the X (transverse) and Y (radial) profiles using the different correction methods for a $38\times 28\;{\text{cm}}^{2}$ field. The $20\times 20\;{\text{cm}}^{2}$ food field correction method was not used for the $38\times 28\;{\text{cm}}^{2}$ field because it was unsuitable for fields larger than $20\times 20\;{\text{cm}}^{2}$. Figures 5(a) and 5(b) give the X and Y profiles from the different correction methods for a $10\times 10\;{\text{cm}}^{2}$ field, including the $20\times 20\;{\text{cm}}^{2}$ food field correction method. For the X (transverse) profiles in 4, 5 there was not a considerable difference between the corrected profiles using either our in‐house method with a $40\times 30\;{\text{cm}}^{2}$ or $20\times 20\;{\text{cm}}^{2}$ food field or the Varian PDPC method.

The $40\times 30\;{\text{cm}}^{2}$ food field calibration includes more backscatter than typical small fields used in IMRT, which manifests as an underresponse of the detector on the gantry side for smaller fields, as seen in the uncorrected Y (radial) profile in Fig. 5(b). None of the three correction methods fully compensates for this asymmetry. For fields comparable to the food field size, as shown in Fig. 4(b), the uncorrected profile does not exhibit any asymmetry. What can be observed in Fig. 4(b) is that the uncorrected profile does exhibit an overresponse at large off‐axis distances when compared to the PDIP predicted profile. Our in‐house 1D method corrects for most of this overresponse without introducing any asymmetry, while the Varian PDPC correction method does not perform well for this field size and introduces an asymmetry into the profile.

From Van Esch et al.^(24)^ and the PDPC customer release notes,^(25)^ this asymmetry as seen in Fig. 4(b) with the Varian PDPC method is explained by the fact that a compromise was made to improve the backscatter correction for field sizes up to $15\times 15\;{\text{cm}}^{2}$, but for larger fields, the disagreements between the predicted and measured profiles can be up to 3%. However, when using a smaller field size, such as that used in IMRT or RapidArc, the differences in the Y profile between the three correction methods is minimal, as seen in Fig. 5(b).

Table 1 gives the average, minimum, and maximum gamma pass rates for the different QA plans delivered using the different profile correction methods, which can be used to compare representative portal dosimetry results against typical QA results using other measurement methods. For the square fields, the uncorrected method had low passing rates when compared to the other three correction methods. The $40\times 30\;{\text{cm}}^{2}$ food field correction method had gamma pass rates that were slightly better than the Varian PDPC method for field sizes under $28\times 28\;{\text{cm}}^{2}$.

With all methods of calibrating the EPID, the maximum difference in the average gamma pass rates for the IMRT fields delivered at gantry 0°, and those delivered at the original planned angles indicates that there is no gravitational problem with using the portal imager for fixed‐field IMRT or RapidArc deliveries.

For the RapidArc plans, neither the $40\times 30\;{\text{cm}}^{2}$ food field correction method nor the Varian PDPC correction method produced an average gamma pass rate larger than the uncorrected EPID calibration method. There was not much room for improvement since the average gamma pass rates for the RapidArc plans without any correction were already quite high.

Even though it was theorized that the smaller food field size ($20\times 20\;{\text{cm}}^{2}$) would present a less asymmetric response, this is not evident in the results of Table 1. This is because the $20\times 20\;{\text{cm}}^{2}$ food field correction method reduces the usable EPID area smaller than the field size being measured for some of our larger split‐field IMRT plans (i.e., the X1, X2, Y1, or Y2 dimensions are greater than 10 cm) resulting in poor gamma pass rates, as seen in Table 1. However, as seen by the profile comparisons, this method could be considered if one is only using field sizes up to a symmetric $20\times 20\;{\text{cm}}^{2}$. Further analysis, which leaves out 8 of the 23 fields tested which violate this criterion, shows that the average, minimum, and maximum gamma pass rates for fields delivered at gantry 0 were 96.8%, 92.2%, and 99.7%, respectively, and the results for fields delivered at the planned gantry angles were 97.0%, 92.6%, and 99.7%, respectively.

**Table 1:** The average, minimum, and maximum gamma pass rates for different QA plans delivered using the different profile correction methods

	*Average Gamma Pass Rate*	*Minimum Gamma Pass Rate*	*Maximum Gamma Pass Rate*	*Std Dev Gamma Pass Rate*	*# of Fields*
*Uncorrected*					
Square Fields (<28×28)	89.4	74.8	95.7	9.02	5
IMRT delivered at Gantry 0	97.7	93.3	99.8	2.05	23
IMRT delivered at planned gantry angle	97.8	93.6	99.7	1.98	23
RapidArc	99.8	99.5	100	0.26	4
*1D Profile Correction (* 40×30 *profile correction method* w/40×30 *food field)*					
Square Fields (<28×28)	95.7	94.7	96.4	0.71	5
IMRT delivered at Gantry 0	98.1	93.7	99.8	2.02	23
IMRT delivered at planned gantry angle	98.1	93.8	99.8	1.94	23
RapidArc	99.6	99.4	99.9	0.22	4
*1D Profile Correction* (20×20 *profile correction method* w/20×20 *food field)*					
Square Fields (<20×20)	91.3	79.8	96.5	7.73	4
IMRT delivered at Gantry 0	92.8	79.8	99.7	6.78	23
IMRT delivered at planned gantry angle	92.8	79.2	99.7	6.83	23
RapidArc	97.0	96.0	98.0	1.02	4
*Varian PDPC Method*					
Square Fields (<28×28)	95.3	93.0	97.6	1.84	5
IMRT delivered at Gantry 0	98.4	93.1	99.8	1.81	23
IMRT delivered at planned gantry angle	98.2	94.1	99.7	1.86	23
RapidArc	99.8	99.5	99.9	0.19	4

One interesting point was that in order to work, our Varian PDPC correction required using the aS500 files available in the package, even though our EPID model is aS1000. It is unclear why this was the case other than the fact that our EPIDs are half sampled, and it is also undetermined at this point as to what uncertainties are associated with this difference.

More recently Bailey et al.^(28)^ presented a 2D correction method that seems similar to the Varian PDPC method. It would be useful to have a comparison between their methods and the Varian PDPC method. However, Bailey and colleagues indicate that their method includes in‐house software, which makes it difficult to implement widely.

## IV. CONCLUSIONS

We have determined that Varian's PDPC correction method produces similar results to our 1D in‐house correction method using the $40\times 30\;{\text{cm}}^{2}$ food field in terms of overall gamma pass rates. We will possibly implement the Varian PDPC correction method for our 21EX portal imagers. However, our analysis demonstrates that, for our other Varian linac models, our 1D correction method should suffice for any necessary QA measurements until a more universal method of applying 2D corrections is available.

## References

[1] Van Esch A , Depuydt T , Huyskens DP . The use of an aSi‐based EPID for routine absolute dosimetric pre‐treatment verification of dynamic IMRT fields. Radiother Oncol. 2004;71(2):223–34. 15110457 10.1016/j.radonc.2004.02.018

[2] Van Esch A , Huyskens DP , Behrens CF , et al. Implementing RapidArc into clinical routine: a comprehensive program from machine QA to TPS validation and patient QA. Med Phys. 2011;38(9):5146–66. 21978060 10.1118/1.3622672

[3] Greer PB and Barnes MP . Investigation of an amorphous silicon EPID for measurement and quality assurance of enhanced dynamic wedge. Phys Med Biol. 2007;52(4):1075–87. 17264371 10.1088/0031-9155/52/4/014

[4] Howell RM , Smith IP , Jarrio CS . Establishing action levels for EPID‐based QA for IMRT. J Appl Clin Med Phys. 2008;9(3):16–25. 10.1120/jacmp.v9i3.2721 PMC5722294 18716584

[5] Nicolini G , Vanetti E , Clivio A , et al. The GLAaS algorithm for portal dosimetry and quality assurance of RapidArc, an intensity modulated rotational therapy. Radiat Oncol. 2008;3(1):24–34. 18782447 10.1186/1748-717X-3-24 PMC2553075

[6] Nicolini G , Vanetti E , Clivio A , Fogliata A , Boka G , Cozzi L . Testing the portal imager GLAaS algorithm for machine quality assurance. Radiat Oncol. 2008;3(1):14–34. 18495005 10.1186/1748-717X-3-14 PMC2430969

[7] Fredh A , Korreman S , Munck af Rosenschöld P . Automated analysis of images acquired with electronic portal imaging device during delivery of quality assurance plans for inversely optimized arc therapy. Radiother Oncol. 2010;94(2):195–98. 20122748 10.1016/j.radonc.2010.01.002

[8] Iori M , Cagni E , Paiusco M , Munro P , Nahum AE . Dosimetric verification of IMAT delivery with a conventional EPID system and a commercial portal dose image prediction tool. Med Phys. 2010;37(1):377–90. 20175500 10.1118/1.3271107

[9] Bakhtiari M , Kumaraswamy L , Bailey DW , de Boer S , Malhotra HK , Podgorsak MB . Using an EPID for patient‐specific VMAT quality assurance. Med Phys. 2011;38(3):1366–73. 21520847 10.1118/1.3552925

[10] Fogliata A , Clivio A , Fenoglietto P , et al. Quality assurance of RapidArc in clinical practice using portal dosimetry. Br J Radiol. 2011;84(1002):534–45. 21606069 10.1259/bjr/72327299 PMC3473641

[11] Jørgensen MK , Hoffmann L , Petersen JB , Præstegaard LH , Hansen R , Muren LP . Tolerance levels of EPID‐based quality control for volumetric modulated arc therapy. Med Phys. 2011;38(3):1425–34. 21520854 10.1118/1.3552922

[12] King BW , Clews L , Greer PB . Long‐term two‐dimensional pixel stability of EPIDs used for regular linear accelerator quality assurance. Australas Phys Eng Sci Med. 2011;34(4):459–66. 22038292 10.1007/s13246-011-0106-0

[13] Bailey DW , Kumaraswamy L , Podgorsak MB . A fully electronic intensity‐modulated radiation therapy quality assurance (IMRT QA) process implemented in a network comprised of independent treatment planning, record and verify, and delivery systems. Radiol Oncol. 2010;44(2):124–30. 22933903 10.2478/v10019-010-0017-9 PMC3423679

[14] Mancuso GM , Fontenot JD , Gibbons JP , Parker BC . Comparison of action levels for patient‐specific quality assurance of intensity modulated radiation therapy and volumetric modulated arc therapy treatments. Med Phys. 2012;39(7):4378–85. 10.1118/1.4729738 22830770

[15] Ko L , Kim JO , Siebers JV . Investigation of the optimal backscatter for an aSi electronic portal imaging device. Phys Med Biol. 2004;49(9):1723–38. 15152927 10.1088/0031-9155/49/9/010

[16] Greer PB , Cadman P , Lee C , Bzdusek K . An energy fluence‐convolution model for amorphous silicon EPID dose prediction. Med Phys. 2009;36(2):547–55. 19291994 10.1118/1.3058481

[17] Berry SL , Polvorosa CS , Wuu C‐S . A field size specific backscatter correction algorithm for accurate EPID dosimetry. Med Phys. 2010;37(6):2425–34. 10.1118/1.3400043 20632552

[18] Rowshanfarzad P , Sabet M , O'Connor DJ , Greer PB . Reduction of the effect of non‐uniform backscatter from an E‐type support arm of a Varian a‐Si EPID used for dosimetry. Phys Med Biol. 2010;55(22):6617–32. 20962364 10.1088/0031-9155/55/22/003

[19] Rowshanfarzad P , McCurdy BM , Sabet M , Lee C , O'Connor DJ , Greer PB . Measurement and modeling of the effect of support arm backscatter on dosimetry with a Varian EPID. Med Phys. 2010;37(5):2269–78. 20527561 10.1118/1.3369445

[20] Vinall AJ , Williams AJ , Currie VE , Van Esch A , Huyskens D . Practical guidelines for routine intensity‐modulated radiotherapy verification: pre‐treatment verification with portal dosimetry and treatment verification with in vivo dosimetry. Br J Radiol. 2010;83(995):949–57. 20965905 10.1259/bjr/31573847 PMC3473728

[21] Bailey DW , Kumaraswamy L , Podgorsak MB . An effective correction algorithm for off‐axis portal dosimetry errors. Med Phys. 2009;36(9):4089–94. 19810481 10.1118/1.3187785

[22] Mans A , Wendling M , McDermott LN , et al. Catching errors with in vivo EPID dosimetry. Med Phys. 2010;37(6):2638–44. 10.1118/1.3397807 20632575

[23] Fuangrod T , Woodruff HC , van Uytven E , et al. A system for EPID‐based real‐time treatment delivery verification during dynamic IMRT treatment. Med Phys. 2013;40(9):091907. 24007158 10.1118/1.4817484

[24] Van Esch A , Huyskens DP , Hirschi L , Baltes C . Optimized Varian aSi portal dosimetry: development of datasets for collective use. J Appl Clin Med Phys. 2013;14(6):82–99. 10.1120/jacmp.v14i6.4286 PMC5714635 24257272

[25] Varian Medical Systems . Portal dosimetry pre‐configuration package. Report No.: PDPC1000RN. Palo Alto, CA: Varian Medical Systems; 2012.

[26] Varian Medical Systems . Installation and verification of the portal dosimetry pre‐configuration package 1.0. Report No.: PV‐887. Palo Alto, CA: Varian Medical Systems; 2012.

[27] Greer PB . Correction of pixel sensitivity variation and off‐axis response for amorphous silicon EPID dosimetry. Med Phys. 2005;32(12):3558–68. 16475754 10.1118/1.2128498

[28] Bailey DW , Kumaraswamy L , Bakhtiari M , Podgorsak MB . A two‐dimensional matrix correction for off‐axis portal dose prediction errors. Med Phys. 2013;40(5):051704. 23635252 10.1118/1.4800493

